# Theoretical Analysis of Buckling for Functionally Graded Thin Plates with Microstructure Resting on an Elastic Foundation

**DOI:** 10.3390/ma13184031

**Published:** 2020-09-11

**Authors:** Jarosław Jędrysiak, Magda Kaźmierczak-Sobińska

**Affiliations:** Department of Structural Mechanics, Łódź University of Technology, al. Politechniki 6, 90-924 Łódź, Poland; jarek@p.lodz.pl

**Keywords:** functionally graded thin plate, tolerance modelling, effect of microstructure

## Abstract

In this paper, the problem of the stability of functionally graded thin plates with a microstructure is presented. To analyse this problem and take into consideration the effect of microstructure, tolerance modelling is used. The tolerance averaging technique allows us to replace the equation with non-continuous, tolerance-periodic, highly oscillating coefficients of the system of differential equations with slowly-varying coefficients, which describes also the effect of the microstructure. As an example, the buckling of a microstructured functionally graded plate band on a foundation is investigated. To obtain results, the tolerance model and the asymptotic model combined together with the Ritz method are used. It is shown that the tolerance model allows us to take into account the effect of microstructure on critical forces.

## 1. Introduction

**Figure 1:**
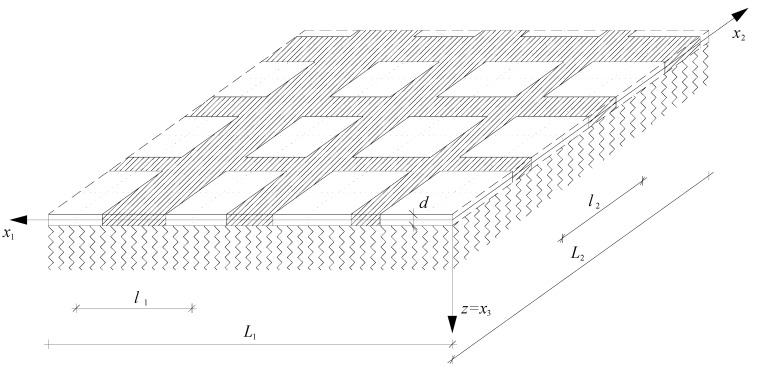
A fragment of a microstructured functionally graded plate (tolerance-periodic plate) on a heterogeneous elastic foundation.

The stability problem of thin functionally graded microstructured plates is considered in this paper. Moreover, these plates can interact with a heterogeneous elastic foundation. It is assumed that on the macrolevel, the plate has functionally graded distributions of properties in planes parallel to the plate midplane, but on the microlevel has a tolerance-periodic structure in these planes, cf. References [1,2]. This structure of the plate can be caused by a proper distribution of geometric properties (a plate thickness) or material properties (mixture/system of various materials). Hence, plates of this kind can be called as *functionally graded* or also as *tolerance-periodic plates*. In such plates, a “basic cell” can be marked, which can be treated as a thin plate, cf. Figure 1, with a fragment of the plate. These adjacent cells are almost identical, but distant ones can be very different.

Functionally graded structures are often described using the known averaging approaches, proposed for periodic media. Some of them can be found in Reference [1]. In the modelling of functionally graded microstructured plates, similar methods can be used. Models based on the asymptotic homogenization approach, cf. Reference [3], can be mentioned, e.g., applied to analyse periodic plates, cf. Reference [4]. To describe different composite media (also periodically microstructured) there are also proposed various other modelling methods, e.g., a homogenization based on microlocal parameters for periodic plates was used in Reference [5]; natural frequencies of thick plates made of orthotropic materials were analysed in Reference [6]; the stability of thin-walled columns with cells was considered in Reference [7]; the dynamic stability of sandwich beams and plates with a core having variable mechanical properties was analysed in References [8,9,10,11]; the stability of three-layered annular plates composed of laminated fibre-reinforced composite facings and foam core was investigated in Reference [12]; a torsion of composite beams with a phase made of an auxetic material with a microstructure was analysed in References [13,14], applying a certain analytical-numerical model based on analytical relations and the finite element method.

Different theoretical and numerical results of various problems of functionally graded structures can be found in many articles. The higher-order theory for various thermomechanical problems of functionally graded fibre reinforced composites with microstructure was proposed and developed in Reference [15,16,17,18]. Applications of the known numerical methods for functionally graded materials were shown in many papers, e.g., the boundary element method was applied to the thermal analysis of composites with fibres in Reference [19]; a certain implementation of the finite element method for functionally graded materials was presented in Reference [20]. The stability of cylindrical shells with functionally graded structure applying Donnell type dynamic stability equations was analysed in Reference [21]. Meshless methods were used to investigate natural frequencies in a few articles, e.g., in Reference [22] for functionally graded plates, and in Reference [23] for sandwich beams with the functionally graded structure of a core. Higher-order plate theories and a collocation method were applied to vibrations of functionally graded plates in Reference [24]. A GDQ solution was proposed in Reference [25] to analyse the free vibration problems of shells. Higher-order deformation theories were applied to analyse thermomechanical problems of functionally graded plates and shells in Reference [26,27]. The statics of doubly-curved functionally graded shells was considered in Reference [28,29,30]. The non-classical FGM plate model based on the modified couple stress theory was proposed for the thermal buckling of annular functionally graded plates in Reference [31], where the size effect, related to the couple stress theory, was analysed. The influence of the shear correction function in a modal analysis of functionally graded beams was investigated in Reference [32]. An optimization of free vibrations for functionally graded beams was presented in Reference [33]. Free vibrations of thick functionally graded plates were considered in Reference [34], taking into account the effects of normal and shear deformations. A higher-order normal and shear deformable theory of plates was used in Reference [35] for vibrations of rectangular functionally graded plates. Non-linear analysis based on a shear deformation theory for functionally graded plates was presented in Reference [36]. A chaos problem for a rectangular functionally graded plate was investigated in Reference [37]. A strong formulation based on the GDQ technique to finite element method for multilayered plates was proposed in Reference [38], but a strong formulation of isogeometric analysis for composite laminated plates was shown in Reference [39]. A differential quadrature method and a layer-wise theory for composite plates were applied in Reference [40]. A new low-order shell element was used in Reference [41] for shell structures having functionally graded material properties. The differential quadrature method was applied to analyse different problems of functionally graded shells and, plates e.g., in Reference [42] for natural frequencies of sandwich shells; for dynamic stability of layered shells in Reference [43]. The theory of sinusoidal shear deformation was applied in Reference [44,45] to describe the bending of piezoelectric functionally graded plates on a foundation and to analyse the free vibrations of composite functionally graded polymer nanoplates. The classical laminate plate theory was applied to formulate the semi-analytical method to analyse certain stability and dynamical problems of thin functionally graded plates, cf. Reference [46], or of columns with open/closed cross-sections made of those plates, cf. Reference [47,48]. Free vibrations of thermally loaded functionally graded sandwich plates were investigated using three-dimensional finite element modelling in Reference [49]. The transient behaviors of functionally graded materials plates with the influence of in-plane displacements and of temperature changes were considered in Reference [50] applying a new semi-analytical algorithm. In Reference [51], an analytical method was proposed using the complex variable approach to analyse forces and moments acting on infinite symmetric functionally graded plates with a triangular hole. Love waves propagated in a functionally graded saturated layer resting on a saturated semi-space were considered in Reference [52]. A certain review of modelling methods for plates with functional gradation of material properties is shown in Reference [53]. However, the analysed examples concerned the change of properties along the thickness of the plate, and not in the midplane of the plate as in this paper. 

These models are described by governing equations, which usually vanish the effect of microstructure size, cf. Reference [54], and this effect cannot be analysed by these models. However, this effect may be relevant to the overall behaviour of microheterogeneous structures. This can be observed in a few of articles, e.g., a spectral element method was used in Reference [55] to analyse the properties of the vibration bandgap for Mindlin’s periodic plates; a centre finite difference method was applied in Reference [56,57] to investigate band gaps of thin periodic plates with or without damping; the differential quadrature element method was used in Reference [58] to analyse flexural wave band gaps in periodic composite plates. 

The effect of microstructure size can be also analysed using *the tolerance averaging method*, cf. Reference [2,59,60]. This modelling approach allows us to include this effect in the governing equations, which describe the overall behaviour of media with a microstructure, which can be caused by the distribution of different materials. This method makes it possible to investigate various dynamical, stability, and thermoelastic problems for periodic structures, which can be found in a series of articles. For instance: fluid-saturated periodic grounds were investigated in Reference [61]; the dynamics of periodic plane structures was considered in Reference [62]; the vibrations of wavy-type periodic plates were analysed in Reference [63]; the dynamics of periodic thin plates reinforced by stiffeners was described in Reference [64]; an application for vibrations of medium thickness periodic plates was shown in Reference [65]; a stability of periodic thin plates on a foundation was considered in Reference [66]; the dynamics of thin periodic plates with the microstructure size of an order of the thickness of the plate was analysed in Reference [67]; applications for dynamics and stability of shells with a periodic microstructure were presented in References [68,69]; the nonlinear dynamics of periodic visco-elastic plates was shown in Reference [70]; the geometrically nonlinear dynamics of periodic beams was analysed in Reference [71,72]; the vibrations of three-layered periodic plates were considered in Reference [73]; an analysis of free vibrations for thin periodic plates having uncertain material properties was presented in Reference [74].

The tolerance modelling approach can be also successfully applied to consider various problems of thermomechanics for functionally graded structures. For instance: investigations of dynamics for longitudinally graded plates were presented in References [75,76,77]; vibrations of thin functionally graded plates with the size of the microstructure of an order of the thickness of the plate were analysed in Reference [78]; vibrations of thin transversally graded plates with the thickness smaller than the microstructure size were shown in Reference [2,79,80]; dynamics of a thin-walled structure with a system of ribs was presented in Reference [81,82]; heat distribution of composite cylindrical conductors having non-uniform microstructure was analysed in Reference [83,84]; problems of thermoelasticity in transversally graded laminates were considered in Reference [85]; dynamics of thin functionally graded microstructured shells was presented in Reference [86,87,88].

On the basis of the literature, it can be noted that the vast majority of the analysed mechanical problems concern layered functionally graded beams, plates, shells, or such structures with functional gradation along the thickness. Only a relatively small part of the work is devoted to the mechanical problems of structures with functional graded properties along the length, i.e., in the plane of the plate, in the shell surface, and along the longitudinal axis of the beam/bar. It seems that most of them show models based on the tolerance modelling method, cf. References [2,60,75,76,77,78,79,80,81,82,86,87,88], and between them References [76,88] present stability problems of functionally graded plates or shells.

In this contribution, *the tolerance model equations* of the thin functionally graded microstructured plates with in-plane forces, taking into account the effect of microstructure size, are presented. Moreover, in order to evaluate the obtained results, the asymptotic model equations known from the literature were introduced. These equations of both the models are also applied to analyse the buckling of a simply supported microstructured plate band. To obtain formulas of critical forces, the Ritz method is applied. In this work, an application of the tolerance model to the analysis of critical forces of thin functionally graded plate bands on an elastic foundation is shown, including microstructure size, an elasticity coefficient of the foundation, and different distributions of properties along the length of the plate band. In the presented example, there are considered functionally graded plate bands with a material microstructure related to the proposed different distribution of two materials.

## 2. Modelling Approach

### 2.1. Modelling Preliminaries

Denote by *Ox*_1_ × _2_*x*_3_ the orthogonal Cartesian coordinate system. Let subscripts *i*, *k*, *l* run over 1,2,3, but α, β, γ run over 1,2. Denote also **x** ≡ (*x*_1_,*x*_2_), *z* ≡ *x*_3_. Let the region of the undeformed plate be denoted by Ω ≡ {(x,z): −*d*/2 ≤ z ≤ *d*/2, **x**∈Π}, where Π is the plate midplane and *d*(·) is the plate thickness. Derivatives of *x*_α_ are denoted by *δ*_α_ and also *δ*_α__...__δ_ ≡ *δ*_α_...*δ*_δ_. Let Δ ≡ [−*l*_1_/2, *l*_1_/2] × [−*l*_2_/2, *l*_2_/2] be the “basic cell” on *Ox*_1_*x*_2_, with *l*_α_ as its length dimensions along the *x*_α_-axis. The diameter of cell Δ is denoted by *l ≡* [(*l*_1_)^2^ + (*l*_2_)^2^]^1/2^ and is called *the microstructure parameter*. It is assumed that this parameter satisfies condition *d*_max_ << *l* << min(*L*_1_,*L*_2_). Thickness *d*(·) can be a tolerance-periodic function in **x** and also elastic moduli *a_ijlm_* = *a_ijlm_*(·,*z*) can be tolerance-periodic functions in **x** and even functions in *z*. Denote *c*_αβγδ_ ≡ *a*_αβγδ_−*a*_αβ33_*a*_γδ33_(*a*_3333_)^−1^, *c*_α3γ3_ ≡ *a*_α3γ3_*−a*_α333_*a*_33γ3_(*a*_3333_)*^−^*^1^, where *a*_αβγδ_, *a*_αβ33_, *a*_α3γ3_, *a*_3333_ are the non-zero components of the elastic moduli tensor. We also assume that properties of an elastic foundation of the Winkler’s type are described by a tolerance-periodic function in **x**—a Winkler’s coefficient *k*. Let *w*(**x**) (**x**∈Π) be a plate deflection and *p* be total loadings in the *z*-axis direction.

Denoting bending stiffnesses *d*_αβγδ_ of the plate, being tolerance-periodic functions in **x**, in the form:

(1) $$d_{\alpha \beta \gamma \delta }(x)\equiv \int_{-d/2}^{d/2}c_{\alpha \beta \gamma \delta }(x,z)z^{2}dz,$$

from the Kirchhoff-type plates theory assumptions used to functionally graded plates the lagrangean can be formulated:

(2) $$\Lambda =-\frac{1}{2}(d_{\alpha \beta \gamma \delta }\partial_{\alpha \beta }w\partial_{\gamma \delta }w+\partial_{\alpha }wn_{\alpha \beta }\partial_{\beta }w+kww)+pw,$$

for which the Euler-Lagrange equation can be written:

(3) $$-\partial_{\alpha \beta }\frac{\partial \Lambda }{\partial \;\partial_{\alpha \beta }w}+\partial_{\alpha }\frac{\partial \Lambda }{\partial (\partial_{\alpha }w)}-\frac{\partial \Lambda }{\partial \;w}=0.$$

Combining Equations (2) and (3), the known fourth-order partial differential equation for deflection *w*(**x**) is obtained:

(4) $$\partial_{\alpha \beta }(d_{\alpha \beta \gamma \delta }\partial_{\gamma \delta }w)-\partial_{\beta }(n_{\alpha \beta }\partial_{\alpha }w)+kw=p.$$

Equation (4) has non-continuous, highly oscillating, tolerance-periodic functional coefficients.

### 2.2. Introductory Concepts of the Tolerance Modelling

Certain concepts defined in References [2,60] are applied in the modelling. Hence, some of them are given below, but for functionally graded plates they were also shown in Reference [2,79,80].

Let ∆(**x**) ≡ **x** + ∆ be a cell at **x**∈Π_∆_, Π_∆_ = {**x** ∈ Π: ∆(**x**) ⊂ Π}. The *averaging operator* for an integrable function *f* is defined by

(5) $$<f>(x)=\frac{1}{l_{1}l_{2}}\int_{\Delta (x)}f(y_{1},y_{2})dy_{1}dy_{2},\;x\in \Pi .$$

The averaged value calculated from (5) of function *f*, being a tolerance-periodic in **x**, is a slowly-varying function in **x**.

Let $\partial^{k}f$ denote the *k*-th gradient of function *f* = *f*(x), **x** ∈ Π, *k* = 0, 1, …, α, (α ≥ 0); $\partial^{0}f\equiv f$; and ${\overset{˜}{f}}^{\left(k\right)}(\cdot ,\cdot )$ be a function defined in Π × *R^m^*. Moreover, the parameter δ is small, δ < 1, called *the tolerance parameter*. This parameter is related to and dependent on every problem under consideration.

Function *f* ∈ *H*^α^ (Π) is *the tolerance-periodic function*, $f\in TP_{\delta }^{\alpha }(\Pi ,\Delta )$, if for *k* = 0, 1, …, α, the following conditions are satisfied:

$$\left(\forall x\in \Pi )\;(\exists {\overset{˜}{f}}^{\left(k\right)}(x,\cdot )\in H^{0}(\Delta )\right)[||\partial^{k}f|{}_{\Pi_{\Delta }}(\cdot )-{\overset{˜}{f}}^{\left(k\right)}(x,\cdot )|{|}_{H^{0}(\Pi ,\Delta )}\leq \delta ],\;(1)\int_{\Delta (\cdot )}{\overset{˜}{f}}^{\left(k\right)}(\cdot ,z)dz\in C^{0}(\bar{\Pi }),$$

where function ${\overset{˜}{f}}^{\left(k\right)}(x,\cdot )$ is *the periodic approximation of* $\partial^{k}f$ *in* ∆(**x**), **x** ∈ Π, *k* = 0,1,…,α.

Function *F* ∈ *H*^α^ (Π) is *the slowly-varying function*, $F\in SV_{\delta }^{\alpha }(\Pi ,\Delta )$, if

$$F\in TP_{\delta }^{\alpha }(\Pi ,\Delta ),\;(\forall x\in \Pi )\;[{\tilde{F}}^{\left(k\right)}(x,\cdot ){|}_{\Delta (x)}=\partial^{k}F(x),\;k=0,\ldots ,\alpha ].$$

Function $\varphi \in H^{\alpha }(\Pi )$ is *the highly oscillating function*, $\varphi \in HO_{\delta }^{\alpha }(\Pi ,\Delta )$, if

$$\varphi \in TP_{\delta }^{\alpha }(\Pi ,\Delta ),\;(\forall x\in \Pi )\;[{\overset{˜}{\varphi }}^{\left(k\right)}(x,\cdot ){|}_{\Delta (x)}=\partial^{k}\tilde{\varphi }(x),\;k=0,1,\ldots ,\alpha ].\;\forall \;F\in SV_{\delta }^{\alpha }(\Pi ,\Delta )\exists f\equiv \varphi F\in TP_{\delta }^{\alpha }(\Pi ,\Delta ){\tilde{f}}^{\left(k\right)}(x,\cdot ){|}_{{}_{\Delta (x)}}=F(x)\partial^{k}\tilde{\varphi }(x){|}_{{}_{\Delta (x)}},\;k=1,\ldots ,\alpha .$$

For *k* = 0 let us denote $\overset{˜}{f}\equiv {\overset{˜}{f}}^{\left(0\right)}$. Moreover, for the plates under consideration, the parameter α is restricted to 2, α = 2.

Let *h*(·) be a highly oscillating function, defined on Π, $h\in HO_{\delta }^{2}(\Pi ,\Delta )$, continuous together with gradient *δ*^1^*h*. Gradient *δ*^2^*h* is a piecewise continuous and bounded. Function *h*(·) is *the fluctuation shape function* of the 2-nd kind, $FS_{\delta }^{2}(\Pi ,\Delta )$, if it depends on *l* as a parameter and the conditions hold:

*δ^k^h*∈*O*(*l*^α−*k*^)   for *k* = 0, 1, …, α, α = 2, *δ*^0^*h* ≡ *h*,

<*h*>(**x**) ≈ 0   for every **x**∈Π_∆_.

### 2.3. Tolerance Modelling Assumptions

Applying the introductory concepts, the modelling assumptions can be formulated, cf. References [2,79,80].

*The micro-macro decomposition* is the first assumption, introduced in the form:

(6) $$w(x)=U(x)+h^{A}(x)Q^{A}(x),A=1,\ldots ,M,\;x\in \Pi ,$$

where $U(\cdot ),\;Q^{A}(\cdot )\in SV_{\delta }^{2}(\Pi ,\Delta )$, i.e., they are slowly-varying functions of the 2-nd kind. Functions *U*(·) and *Q^A^*(·) are new basic kinematic unknowns, named *the macrodeflection* and *the fluctuation amplitudes*, respectively; *h^A^*(·) are the known fluctuation shape functions, satisfying the condition <*h^A^h^B^* > ≈ 0, *A*, *B* = 1, …, *M*.

*The tolerance averaging approximation* is the second assumption, in which terms *O*(δ) are assumed to be negligibly small, e.g., for $f\in TP_{\delta }^{2}(\Pi ,\Delta ),$ $F\in SV_{\delta }^{2}(\Pi ,\Delta ),$ $h^{A}\in FS_{\delta }^{2}(\Pi ,\Delta ),$ in:

(7) $$\begin{array}{l} <f>(x)=<\bar{f}>(x)+O(\delta ),\\ <fF>(x)=<f>(x)F(x)+O(\delta ),\\ <f\partial_{\alpha }(h^{A}F)>(x)=<f\partial_{\alpha }h^{A}>(x)F(x)+O(\delta ). \end{array}$$

*The in-plane forces restriction* is the third assumption, which allows us to neglect terms involving fluctuating parts of in-plane forces in comparing to terms with averaged parts, i.e.,:

(8) $$\begin{array}{ll} n_{\alpha \beta }(x)=N_{\alpha \beta }(x)+{\tilde{n}}_{\alpha \beta }(x)\underset{}{,}\\ N_{\alpha \beta }=<n_{\alpha \beta }>, & <{\tilde{n}}_{\alpha \beta }>=0, \end{array}$$

where $N_{\alpha \beta }(\cdot )\in SV_{\delta }^{2}(\Pi ,\Delta )$ and ${\tilde{n}}_{\alpha \beta }(\cdot )\in TP_{\delta }^{2}(\Pi ,\Delta )$ are *averaged* and *fluctuating part of in-plane forces*, respectively.

### 2.4. The Outline of the Tolerance Modelling Procedure

Following References [2] or [79,80], the modelling procedure is briefly introduced. The formulation of the lagrangean Λ in the form (2) is the starting point of the modelling. From combining the Euler-Lagrange equation (3) with the lagrangean (2), the governing equation of the plate is derived in the form (4).

Applying tolerance modelling to the lagrangean (2), there are three steps. In the first, the micro-macro decomposition (6) is substituted into (2); in the second, the averaging operator (5) is used to obtain averaged functional; and in the third, the restriction (8) and the approximation (7) are used for this functional. This leads to the tolerance averaged lagrangean $<L_{h}>$ in the form:

(9) $$\begin{array}{ll} <\Lambda_{h}>=- & \frac{1}{2}\{(<d_{\alpha \beta \gamma \delta }>\partial_{\alpha \beta }U+2<d_{\alpha \beta \gamma \delta }\partial_{\alpha \beta }h^{B}>Q^{B})\partial_{\gamma \delta }U+\\ & +\partial_{\alpha }U<n_{\alpha \beta }>\partial_{\beta }U+<k>UU+2U<kh^{B}>Q^{B}+<kh^{A}h^{B}>Q^{A}Q^{B}+\\ & +(<d_{\alpha \beta \gamma \delta }\partial_{\alpha \beta }h^{A}\partial_{\gamma \delta }h^{B}>+<n_{\alpha \beta }\partial_{\alpha }h^{A}\partial_{\beta }h^{B}>)Q^{A}Q^{B}\}+<p>U+<ph^{A}>Q^{A}. \end{array}$$

From the principle of stationary action applied to (9) the averaged Euler-Lagrange equations for *U*(·,*t*) and *Q^A^*(·,*t*) take the following form:

(10) $$\begin{array}{l} -\partial_{\alpha \beta }\frac{\partial <\Lambda_{h}>}{\partial \;\partial_{\alpha \beta }U}+\partial_{\alpha }\frac{\partial <\Lambda_{h}>}{\partial (\partial_{\alpha }U)}-\frac{\partial <\Lambda_{h}>}{\partial \;U}=0,\\ -\frac{\partial <\Lambda_{h}>}{\partial \;Q^{A}}=0. \end{array}$$

### 2.5. Governing Equations

#### 2.5.1. The Tolerance Model Equations

Substituting the tolerance averaged lagrangean (9) to the averaged Euler-Lagrange equations (10), introducing denotations:

(11) $$\begin{array}{lll} D_{\alpha \beta \gamma \delta }\equiv <d_{\alpha \beta \gamma \delta }>, & D_{\alpha \beta }^{A}\equiv <d_{\alpha \beta \gamma \delta }\partial_{\gamma \delta }h^{A}>, & D^{AB}\equiv <d_{\alpha \beta \gamma \delta }\partial_{\alpha \beta }h^{A}\partial_{\gamma \delta }h^{B}>,\\ K\equiv <k>, & K^{A}\equiv l^{-2}<kh^{A}>, & K^{AB}\equiv l^{-4}<kh^{A}h^{B}>,\\ H_{\alpha \beta }^{AB}\equiv l^{-2}<\partial_{\alpha \beta }h^{A}h^{B}>, & P\equiv <p>, & P^{A}\equiv l^{-2}<ph^{A}>, \end{array}$$

and after some manipulations the following equations for *U*(·) and *Q^A^*(·) are obtained:

(12) $$\begin{array}{l} \partial_{\alpha \beta }(D_{\alpha \beta \gamma \delta }\partial_{\gamma \delta }U+D_{\alpha \beta }^{A}Q^{A})-\partial_{\alpha }(N_{\alpha \beta }\partial_{\beta }U)+KU+\underline{l^{2}K^{A}Q^{A}}=P\underset{}{,}\\ D_{\gamma \delta }^{A}\partial_{\gamma \delta }U+D^{AB}Q^{B}-\underline{l^{2}N_{\alpha \beta }H_{\alpha \beta }^{AB}Q^{B}}+\underline{l^{2}K^{A}U}+\underline{l^{4}K^{AB}Q^{B}}=\underline{l^{2}P^{A}}, \end{array}$$

which stand for the system of the partial differential equations. All coefficients of Equations (12) are slowly-varying functions in **x** in contrast to Equation (2), having non-continuous, highly oscillating, and tolerance-periodic coefficients. The underlined terms in the above equations are dependent of the microstructure parameter *l*. Equations (12) with micro-macro decomposition (6) describe the stability of plates under consideration in the framework of *the tolerance model of thin microstructured functionally graded plates*. These governing equations involve coefficients (underlined) describing the effect of microstructure size in these plates. Hence, this model allows us to analyse this effect in the stability problems of the considered plates. The basic kinematic unknowns *U*, *Q^A^*, *A* = 1,…, *M*, are slowly-varying functions in **x**. Boundary conditions can be only formulated for *the macrodeflection U*, but not for *the fluctuation amplitudes Q^A^*.

#### 2.5.2. The Asymptotic Model Equations

Results obtained in the framework of the tolerance model can be evaluated using an approximate model, in which the effect of microstructure size is neglected. Using the proper asymptotic modelling procedure, cf. References [60,78,79], or after neglecting the underlined terms in Equation (12), the following equation can be written:

(13) $$\partial_{\alpha \beta }(D_{\alpha \beta \gamma \delta }^{eff}\partial_{\gamma \delta }U)-\partial_{\alpha }(N_{\alpha \beta }\partial_{\beta }U)+KU=P,$$

with the effective averaged bending stiffness defined by:

(14) $$D_{\alpha \beta \gamma \delta }^{eff}\equiv D_{\alpha \beta \gamma \delta }-D_{\alpha \beta }^{A}{\left(D^{AB}\right)}^{-1}D_{\gamma \delta }^{B}.$$

Equation (13) represents *the asymptotic model of thin microstructured functionally graded plates*, which neglects the effect of microstructure size. In comparison with Equation (2), which has non-continuous, tolerance-periodic coefficients, Equation (13) has smooth, slowly-varying coefficients. It should be noted that only one differential Equation (13) for the macrodeflection *U*, with the effective stiffness (14), is obtained.

## 3. Application—A Buckling Problem of Simply Supported Functionally Graded Plate Bands

### 3.1. Introduction

**Figure 2:**
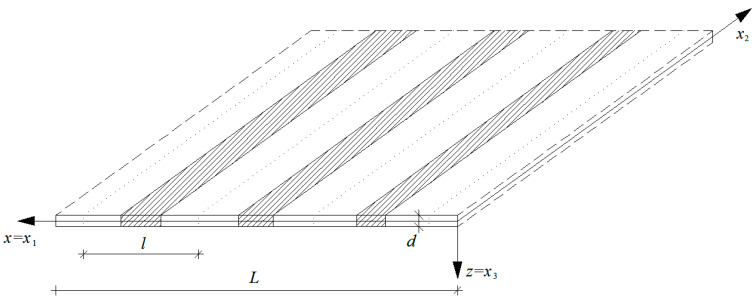
A part of a microstructured functionally graded plate band.

A buckling of a thin plate band with span *L* along the *x*_1_-axis is considered as an example. Thus, the loading *p* is vanished; *p* = 0. It is assumed that a material structure of the plate band is functionally graded along its span, cf. Figure 2. The material properties of the plate are independent of the *x*_2_-coordinate, hence the stability problem under consideration is treated as independent of the *x*_2_-coordinate too.

Let *x* = *x*_1_, *z* = *x*_3_, *x*∈[0,*L*], *z*∈[−*d*/2,*d*/2], where *d* is a constant plate thickness, and *δ* ≡ *δ*_1_. Thus, for this plate, the basic cell is defined as ∆ ≡ [−*l*/2,*l*/2] in the interval Ξ ≡ [0,*L*], and *l* is the length of this cell, *l*<<*L*. Moreover, a cell with a centre at *x*∈[0,*L*] is denoted by ∆(*x*) ≡ [*x*−*l*/2,*x* + *l*/2].

It is assumed that the plate band is made of two-component elastic isotropic materials, described by Young’s moduli *E*′, *E*′′, and Poisson’s ratios ν′, ν′′, respectively. These components are perfectly bonded on interfaces. Let us assume that *E*(·) is a tolerance-periodic function in *x*, $E(\cdot )\in TP_{\delta }^{0}(\Xi ,\Delta )$, but Poisson’s ratio ν ≡ ν′ = ν′′ is constant. The material structure of the plate band can be treated as functionally graded on the macro-scale, and at the same time tolerance-periodic on the micro-scale along the *x*-axis if the condition *E*′ ≠ *E*′′ is satisfied. Moreover, it is assumed that the considered plate band rests on an elastic foundation described by a constant Winkler’s coefficient *k*.

**Figure 3:**
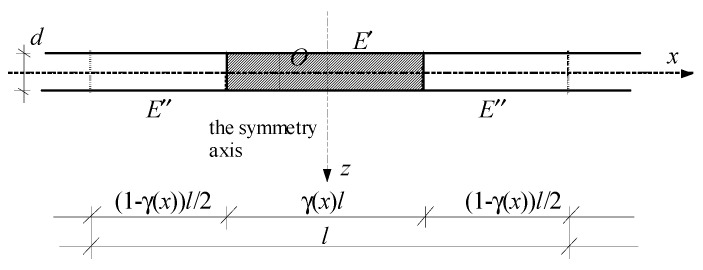
A cell of the microstructured functionally graded plate band.

Let the following function describe the Young’s modulus of the plate band:

(15) $$E(x,y)=\left\{ \begin{array}{ll} E^{\prime }, & \mathrm{for}\;y\in ((1-\gamma (x))l/2,(1+\gamma (x))l/2),\\ E^{\prime \prime }, & \mathrm{for}\;y\in [0,(1-\gamma (x))l/2]\cup [(1+\gamma (x))l/2,l], \end{array}\right.$$

with γ(*x*), *x*∈ Ξ = [0,*L*], as a distribution function of material properties, cf. Figure 3.

In the considerations, let us assume only one fluctuation shape function, i.e., *A* = *M* = 1. Hence, denote *h* ≡ *h*^1^, *Q* ≡ *Q*^1^. Micro-macro decomposition (6) of the field *w*(*x*) takes the form:

(16) w(x)=U(x)+h(x)Q(x),

where $U(\cdot ),\;Q(\cdot )\in SV_{\delta }^{2}(\Xi ,\Delta )$, $h(\cdot )\in FS_{\delta }^{2}(\Xi ,\Delta )$.

For the cell assumed in the form shown in Figure 3, the periodic approximation of the fluctuation shape function *h*(*x*) can have the form:

(17) $$\tilde{h}(x,y)=l^{2}\cos(2\pi y/l),y\in \Delta (x),\;x\in \Xi .$$

Derivatives $\partial \tilde{h},\;\partial \partial \tilde{h}$ of the above function take the form:

(18) $$\partial h(y)=-2\pi l\sin(2\pi y/l),\partial \partial h(y)=-4\pi^{2}\cos(2\pi y/l).$$

Let us assume that the in-plane forces are only along the *x*-axis. Hence, *N*_12_ = *N*_21_ = *N*_22_ = 0, *N*_11_≠0. Denote also *N* = −*N*_11_. Now, tolerance model Equations (12) of the considered plate band have the form:

(19) $$\begin{array}{l} \partial \partial (D_{1111}\partial \partial U+D_{11}^{1}Q)+\partial (N\partial U)+KU+\underline{l^{2}K^{1}Q}=0,\\ D_{11}^{1}\partial \partial U+D^{11}Q+\underline{l^{2}NH_{11}^{11}Q}+\underline{l^{2}K^{1}U}+\underline{l^{4}K^{11}Q}=0. \end{array}$$

Equation (19) describes a stability problem of the thin microstructured functionally graded plate band in the framework of the tolerance model.

Moreover, for the considered plate band of Equation (13) takes the form:

(20) $$\partial \partial (D_{1111}^{eff}\partial \partial U)+\partial (N\partial U)+KU=0,$$

with the effective bending stiffness (14):

(21) $$D_{1111}^{eff}\equiv D_{1111}-D_{11}^{1}{\left(D^{11}\right)}^{-1}D_{11}^{1}.$$

Equation (20) describes a stability problem of this plate band in the framework of the asymptotic model. Equations (19) and (20) have slowly-varying functional coefficients.

### 3.2. The Application of the Ritz Method

Since coefficients of Equations (19) and (20) are slowly-varying functions in *x*, it is difficult to find analytical solutions of them. Hence, formulas of critical forces of plates under consideration can be found applying the known Ritz method, cf. [2,78,79,80]. In this method, formulas of the maximal strain energy W_max_ should be determined.

For the simply supported plate band, the solution to Equations (19) and (20) can be assumed as:

(22) $$U(x)=A_{U}\sin(\alpha x),\;Q(x)=A_{Q}\sin(\alpha x),$$

where: *A_U_* and *A_Q_* are amplitudes of the macrodeflection and the fluctuation amplitude, respectively; α is a wave number, α = *m*π/*L*, with *L* as the length of the plate band, *m* as the number of halfwaves of buckling (*m* = 1, 2, 3, …).

Moreover, denotations of coefficients are introduced:

(23) $$\begin{array}{l} \overset{⌣}{B}=\frac{d^{3}}{12(1-\nu^{2})}\int_{0}^{L}\{E^{\prime \prime }[1-\tilde{\gamma }(x)]+\tilde{\gamma }(x)E^{\prime }\}\sin^{2}(\alpha x)dx,\;\bar{B}=\frac{\pi d^{3}}{3(1-\nu^{2})}(E^{\prime }-E^{\prime \prime })\int_{0}^{L}\sin(\pi \tilde{\gamma }(x))\sin^{2}(\alpha x)dx,\\ \hat{B}=\frac{{\left(\pi d\right)}^{3}}{3(1-\nu^{2})}\int_{0}^{L}\{(E^{\prime }-E^{\prime \prime })[2\pi \tilde{\gamma }(x)+\sin(2\pi \tilde{\gamma }(x))]+2\pi E^{\prime \prime }\}\sin^{2}(\alpha x)dx,\\ G=\int_{0}^{L}\cos^{2}(\alpha x)dx,\;\bar{G}=2\pi^{2}\int_{0}^{L}\sin^{2}(\alpha x)dx,\overset{⌣}{K}=k\int_{0}^{L}\sin^{2}(\alpha x)dx,\;\bar{K}=\frac{1}{2}k\int_{0}^{L}\sin^{2}(\alpha x)dx. \end{array}$$

Using denotations (23) the maximal strain energy W_max_ by the tolerance model takes the form:

(24) $$W_{\max}=\frac{1}{2}[(\overset{⌣}{B}{\left(A_{U}\right)}^{2}\alpha^{2}-2\bar{B}A_{U}A_{Q}-N{\left(A_{U}\right)}^{2}G)\alpha^{2}+\overset{⌣}{K}{\left(A_{U}\right)}^{2}+(\hat{B}+l^{4}\bar{K}-l^{2}N\bar{G}){\left(A_{Q}\right)}^{2}].$$

Applying the conditions of the Ritz method to Equation (24):

(25) $$\frac{\partial W_{\max}}{\partial A_{U}}=0,\;\frac{\partial W_{\max}}{\partial A_{Q}}=0,$$

after some manipulations, the following formulas are obtained:

(26) $$\begin{array}{l} N_{-}\equiv \frac{\bar{G}[{\left(\alpha l\right)}^{2}\overset{⌣}{B}+l^{2}\alpha^{-2}\overset{⌣}{K}]+G(\hat{B}+l^{4}\bar{K})}{2G\bar{G}l^{2}}-\frac{\sqrt{{\left\{\bar{G}[\overset{⌣}{B}{\left(\alpha l\right)}^{2}+l^{2}\alpha^{-2}\overset{⌣}{K}]-G(\hat{B}+l^{4}\bar{K})\right\}}^{2}+4{\left(\alpha l\right)}^{2}G\bar{G}{\bar{B}}^{2}}}{2G\bar{G}l^{2}},\\ N_{+}\equiv \frac{\bar{G}[{\left(\alpha l\right)}^{2}\overset{⌣}{B}+l^{2}\alpha^{-2}\overset{⌣}{K}]+G(\hat{B}+l^{4}\bar{K})}{2G\bar{G}l^{2}}+\frac{\sqrt{{\left\{\bar{G}[\overset{⌣}{B}{\left(\alpha l\right)}^{2}+l^{2}\alpha^{-2}\overset{⌣}{K}]-G(\hat{B}+l^{4}\bar{K})\right\}}^{2}+4{\left(\alpha l\right)}^{2}G\bar{G}{\bar{B}}^{2}}}{2G\bar{G}l^{2}}, \end{array}$$

of *the lower N*_−_ and, so-called, *the higher N*_+_ *critical forces*, respectively, in the framework of *the tolerance model*.

The problem of buckling analysed in the framework of *the asymptotic model* using the Ritz method leads to the following formula:

(27) $$N_{0}\equiv \alpha^{2}\frac{\overset{⌣}{B}\hat{B}-{\bar{B}}^{2}}{G\hat{B}}+\alpha^{-2}\frac{K}{G},$$

of the lower critical force N_0_.

It can be noted that both the tolerance model and the asymptotic model allow us to analyse the problem of buckling for the plate bands under consideration using formulas of the lower (fundamental) critical force (*N*_−_, *N*_0_), but the tolerance model leads also to the formula of the so-called higher critical force (*N*_+_). However, in this paper, considerations are restricted only to the lower critical forces.

### 3.3. Results

Some numerical results will be calculated for four distribution functions of material properties γ(*x*), in which approximations $\tilde{\gamma }(x)$ are assumed in the form:

(28) $$\begin{array}{l} \tilde{\gamma }(x)=\sin^{2}(\pi x/L),\\ \tilde{\gamma }(x)=\cos^{2}(\pi x/L),\\ \tilde{\gamma }(x)={\left(x/L\right)}^{2},\\ \tilde{\gamma }(x)=\sin(\pi x/L), \end{array}$$

and will be compared with results for the periodic plate band describing by the constant distribution function:

(29) $$\tilde{\gamma }(x)=\gamma =0.5,$$

and also for the homogeneous plate band for γ = 0 (the Young’s modulus of the material for the whole plate is *E*’’) and γ = 1 (the Young’s modulus of the material for the whole plate is *E*’).

Let us introduce dimensionless forces ratios given by:

(30) $$n_{-}\equiv \frac{12(1-\nu^{2})}{E^{\prime }L}N_{-},\;n_{0}\equiv \frac{12(1-\nu^{2})}{E^{\prime }L}N_{0},$$

for fundamental critical forces *N*_−_, *N*_0_, described by (26)_1_, (27), respectively.

Moreover, the elastic foundation can be described by dimensionless parameter related to the Winkler’s coefficient in the following form:

(31) $$\kappa =12(1-\nu^{2})kd{\left(E^{\prime }\right)}^{-1}.$$

**Figure 4:**
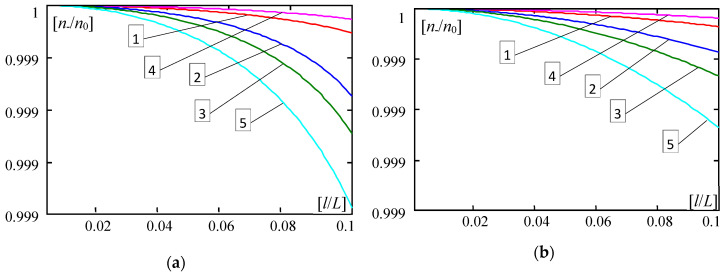
Values of ratios *n*___/*n*_0_ versus *l*/*L* (*E*”/*E*’ = 0.3, *d*/*L* = 0.01, *m* = 1). (**a**) For κ = 0.0001; (**b**) For κ = 0.000001. (1—γ by (28)_1_, 2—γ by (28)_2_, 3—γ by (28)_3_, 4—γ by (28)_4_, 5—γ by (29)).

**Figure 5:**
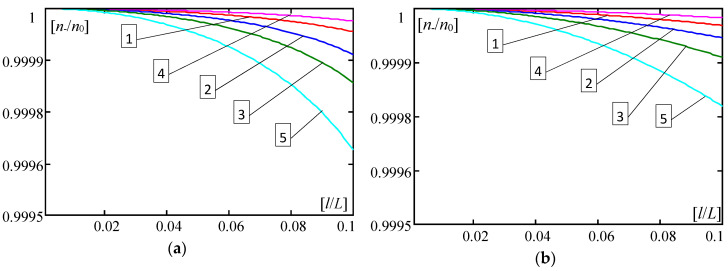
Values of ratios *n*___/*n*_0_ versus *l*/*L* (*E*”/*E*’ = 0.5, *d*/*L* = 0.01, *m* = 1). (**a**) For κ = 0.0001; (**b**) For κ = 0.000001. (1—γ by (28)_1_, 2—γ by (28)_2_, 3—γ by (28)_3_, 4—γ by (28)_4_, 5—γ by (29)).

**Figure 6:**
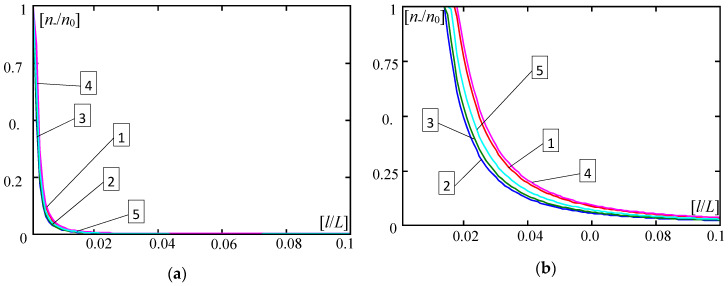
Values of ratios *n*___/*n*_0_ versus *l*/*L* (*E*”/*E*’ = 0.3, *d*/*L* = 0.001, *m* = 1). (**a**) For κ = 0.0001; (**b**) For κ = 0.000001. (1—γ by (28)_1_, 2—γ by (28)_2_, 3—γ by (28)_3_, 4—γ by (28)_4_, 5—γ by (29)).

**Figure 7:**
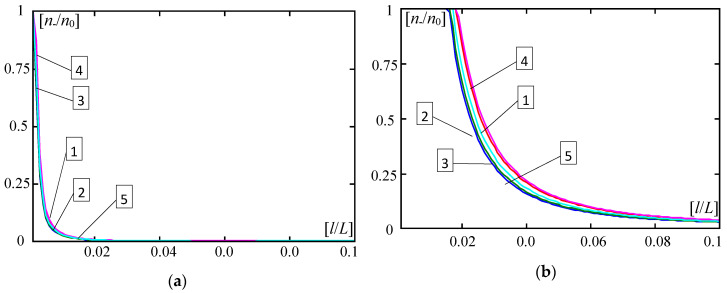
Values of ratios *n*___/*n*_0_ versus *l*/*L* (*E*”/*E*’ = 0.5, *d*/*L* = 0.001, *m* = 1). (**a**) For κ = 0.0001; (**b**) For κ = 0.000001. (1—γ by (28)_1_, 2—γ by (28)_2_, 3—γ by (28)_3_, 4—γ by (28)_4_, 5—γ by (29)).

**Figure 8:**
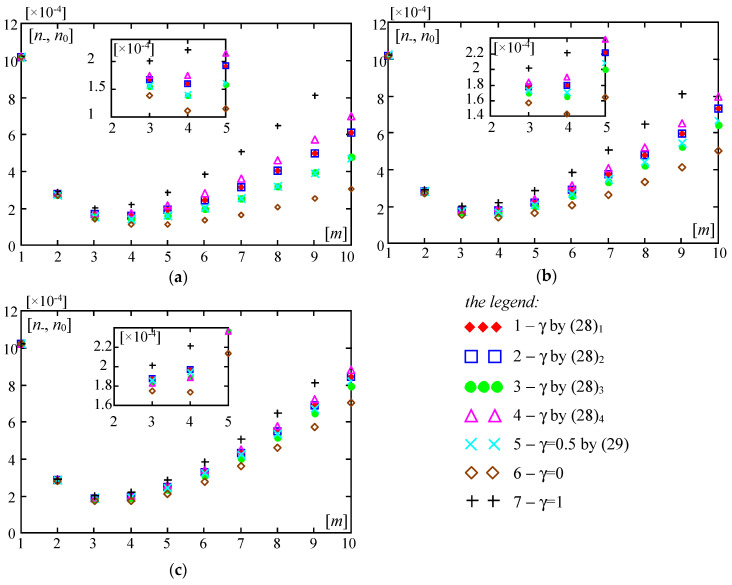
Values of dimensionless parameters *n*___, *n*_0_ of critical forces versus numbers of halfwaves of buckling *m* (*d*/*L* = 0.01, *l*/*L* = 0.1, κ = 10^-4^): (**a**) For *E*′′/*E*′ = 0.3; (**b**) For *E*′′/*E* ′= 0.5; (**c**) For *E*′′/*E*′ = 0.7.

**Figure 9:**
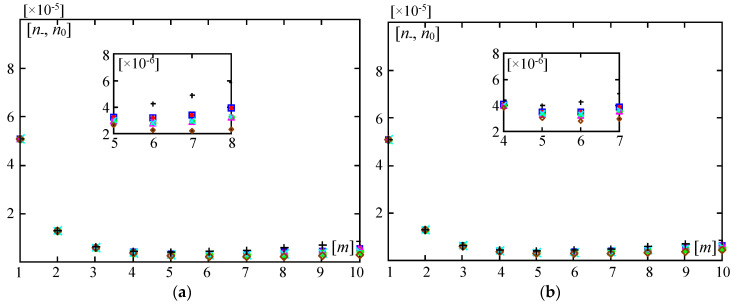

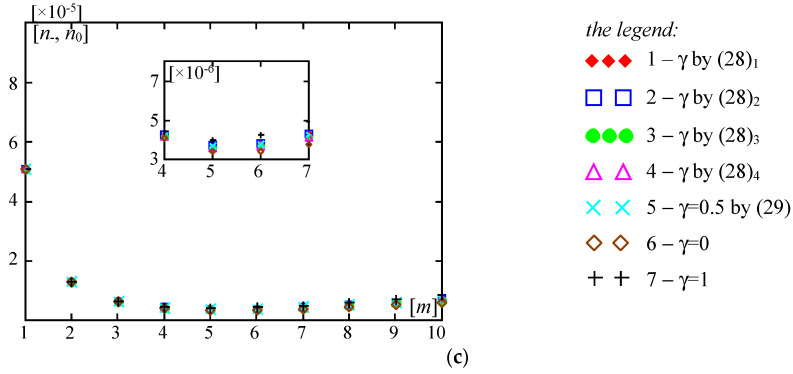
Values of dimensionless parameters *n*___, *n*_0_ of critical forces versus numbers of halfwaves of buckling *m* (*d*/*L* = 0.002, *l*/*L* = 0.02, κ = 10^−6^): (**a**) *E*′′/*E*′ = 0.3; (**b**) *E*′′/*E*′ = 0.5; (**c**) *E*′′/*E*′ = 0.7.

**Figure 10:**
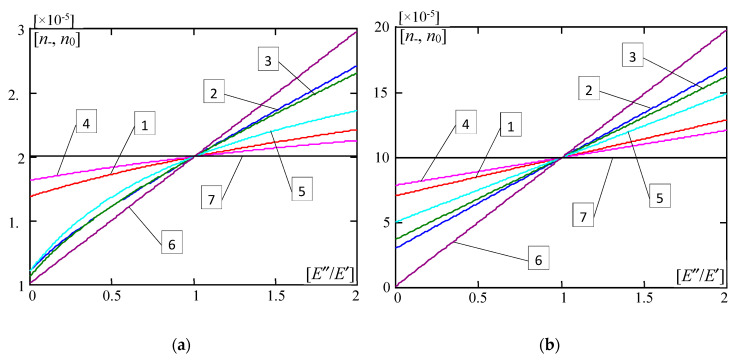
Values of dimensionless parameters *n*___, *n*_0_ of critical forces versus the ratio *E*′′/*E*′ (*l*/*L* = 0.02, *m* = 1): (**a**) *d*/*L* = 0.01, κ = 10^−6^; (**b**) *d*/*L* = 0.001, κ = 10^−4^. (1—γ by (28)_1_, 2—γ by (28)_2_, 3—γ by (28)_3_, 4—γ by (28)_4_, 5—γ by (29)).

Some results calculated by Formula (30) are shown in Figure 4, Figure 5, Figure 6, Figure 7, Figure 8, Figure 9 and Figure 10. These all plots are made for the Poisson’s ratio ν = 0.3. Diagrams in Figure 4, Figure 5, Figure 6 and Figure 7 and Figure 10 are for the number of halfwaves of buckling assumed *m* = 1.

In Figure 4, Figure 5, Figure 6 and Figure 7 there are shown plots of the dimensionless forces ratios *n*_−_/*n*_0_ versus the dimensionless microstructure parameter *l*/*L*∈(0,0.1] for four functionally graded plate bands with the distribution functions of material properties γ(*x*) given by: (28)_1_—curves no. 1, (28)_2_—curves no. 2, (28)_3_—curves no. 3, (28)_4_—curves no. 4; and for the periodic plate band with γ = 0.5, (29), curves no. 5. Plots in Figure 4 and Figure 6 are for the ratio *E”*/*E’* = 0.3; in Figure 5 and Figure 7—for *E”*/*E’* = 0.5. Results in Figure 4 and Figure 5 are obtained for *d*/*L* = 0.01, but in Figure 6 and Figure 7—for *d*/*L* = 0.001. Figure 4, Figure 5, Figure 6 and Figure 7, marked by (a), show curves for the parameter κ = 10^−4^, but marked by (b) show curves for the parameter κ = 10^−6^.

Figure 8 and Figure 9 present diagrams of the dimensionless forces ratios *n*, *n*_0_ versus the number *m* of halfwaves of buckling. They are made for ratios: *d*/*L* = 0.01, *l*/*L* = 0.1, κ = 10^−4^ (Figure 8); *d*/*L* = 0.002, *l*/*L* = 0.02, κ = 10^−6^ (Figure 9). Figure 8 and Figure 9, marked by (a), show curves for the ratio *E”*/*E’* = 0.3, (b) show curves for the ratio *E”*/*E’* = 0.5, and (c) show curves for the ratio *E”*/*E’* = 0.7.

Figure 10 presents plots of the dimensionless forces ratios *n*_−_, *n*_0_ versus the ratio *E”*/*E’*∈(0,2]. They are calculated for parameters: *l*/*L* = 0.02, *m* = 1. Figure 10 marked by (a) shows curves for *d*/*L* = 0.01, κ = 10^−6^; but marked by (b) shows curves for *d*/*L* = 0.001, κ = 10^−4^. Plots in Figure 10 numbered by 1-5 are related to microstructured plate bands described by the distribution functions of material properties γ(*x*) defined by Equations (28) and (29). Moreover, curves numbered by 6 are made for the homogeneous plate band (with γ = 0, i.e., the plate band is made of the material with the Young’s modulus *E”*) and by 7 for the homogeneous plate band (with γ = 1, i.e., the plate band is made of the material with the Young’s modulus *E’*).

## 4. Discussion

Analysing obtained numerical results presented in the above diagrams, it can be observed that differences between critical forces calculated by the tolerance model and by the asymptotic model are small for *d*/*L* ≥ 0.01 (Figure 4 and Figure 5). However, for 0.001 ≥ *d*/*L* (Figure 6 and Figure 7), differences between critical forces can be large. Moreover, these differences between the critical forces for various distribution functions of material properties are bigger for stiffer foundations, e.g., κ = 10^−4^ (Figure 4a, Figure 5a, Figure 6a and Figure 7a) than for softer foundations, e.g., κ = 10^−6^ (Figures 4b, Figure 5b, Figure 6b and Figure 7b).

In Figure 8 and Figure 9, it is shown for what number *m* of halfwaves of buckling a critical force has the smallest value in relation to the distribution function of the material properties. It can be observed that the number *m* for the minimal critical force is related to the ratio *E*′′/*E*′ and distribution functions γ(*x*) ((28), (29)).

For parameters *d*/*L* = 0.01, *l*/*L* = 0.1, κ = 10^−4^ (cf. Figure 8) and for the ratio *E*′′/*E*′ = 0.3, the smallest critical forces for all considered distribution functions γ(*x*) (28) and (29) and for the homogeneous plate band with γ = 0 (i.e., the material of the whole plate has the Young’s modulus *E”*) are obtained for *m* = 4, but only for the homogeneous plate band with γ = 1 (i.e., the material of the whole plate has the Young’s modulus *E’*) the smallest critical force is for *m* = 3. For the ratio *E*′′/*E*′ = 0.5, the smallest critical forces for distribution functions γ(*x*) (28)_3_, (29) and for the homogeneous plate band with γ = 0 (i.e., the material of the whole plate has the Young’s modulus *E”*) are obtained for *m* = 4, but for distribution functions γ(*x*) (28)_1,2,4_ and the homogeneous plate band with γ = 1 (i.e., the material of the whole plate has the Young’s modulus *E’*) the smallest critical force is for *m* = 3. For the ratio *E*′′/*E*′ = 0.7, the smallest critical forces for all considered distribution functions γ(*x*) (28) and (29) and for the homogeneous plate band with γ = 1 (i.e., the material of the whole plate has the Young’s modulus *E’*) are obtained for *m* = 3, but only for the homogeneous plate band with γ = 0 (i.e., the material of the whole plate has the Young’s modulus *E”*) the smallest critical force is for *m* = 4.

For plate bands of smaller thickness, smaller basic cells, and softer foundation, i.e., for parameters: *d*/*L* = 0.002, *l*/*L* = 0.02, κ = 10^−6^ (cf. Figure 9), and for the ratio *E*′′/*E*′ = 0.3, the smallest critical forces for all considered distribution functions γ(*x*) (28) and (29) and for the homogeneous plate band with γ = 1 (i.e., the material of the whole plate has the Young’s modulus *E’*) are obtained for *m* = 6, but only for the homogeneous plate band with γ = 0 (i.e., the material of the whole plate has the Young’s modulus *E”*) the smallest critical force is for *m* = 7. For the ratio *E*′′/*E*′ = 0.5, the smallest critical forces for distribution functions γ(*x*) (28)_3_, (29) and for the homogeneous plate band with γ = 0 (i.e., the material of the whole plate has the Young’s modulus *E”*) they are obtained for *m* = 6, but for distribution functions γ(*x*) (28)_1,2,4_ and the homogeneous plate band with γ = 1 (i.e., the material of the whole plate has the Young’s modulus *E’*) the smallest critical force is for *m* = 5. For the ratio *E*′′/*E*′ = 0.7 the smallest critical forces for all considered distribution functions γ(*x*) (28) and (29) and for the homogeneous plate band with γ = 1 (i.e., the material of the whole plate has the Young’s modulus *E’*) are obtained for *m* = 5, but only for the homogeneous plate band with γ = 0 (i.e., the material of the whole plate has the Young’s modulus *E”*) the smallest critical force is for *m* = 6.

From the results shown in Figure 10, it can be observed that values of dimensionless critical forces ratios for all microstructured plate bands fit between those specified for homogeneous plate bands with γ = 0 (i.e., the material of the whole plate has the Young’s modulus *E”*) and with γ = 1 (i.e., the material of the whole plate has the Young’s modulus *E’*). Linear plots of results are only for these two homogeneous plate bands. All other plots for microstructured plate bands are curvilinear, and it is more visible for thicker plates and a less rigid foundation (for e.g., *d*/*L* = 0.01, κ = 10^−6^; cf. Figure 10a). Moreover, for the ratio *E*′′/*E*′ > 1 values of dimensionless critical forces ratios for all microstructured plate bands are bigger than these ratios for the homogeneous plate band with γ = 1 (i.e., the material of the whole plate has the Young’s modulus *E’*), but smaller than these ratios for the homogeneous plate band with γ = 0 (i.e., the material of the whole plate has the Young’s modulus *E”*). The descending order of values of these ratios for microstructured plate bands in relation to the distribution functions of properties γ(*x*) is as follows: for γ(*x*) by (28)_2_, for γ(*x*) by (28)_3_, for γ(*x*) by (29), for γ(*x*) by (28)_1_, for γ(*x*) by (28)_4_. However, for the ratio *E*′′/*E*′ < 1, these ratios for all microstructured plate bands are bigger than those for the homogeneous plate band with γ = 0, but smaller than those for the homogeneous plate band with γ = 1. The ascending order of values of these critical forces ratios for microstructured plate bands in relation to the distribution functions of properties γ(*x*) is as follows: for γ(*x*) by (28)_2_, for γ(*x*) by (28)_3_, for γ(*x*) by (29), for γ(*x*) by (28)_1_, for γ(*x*) by (28)_4_. However, it should be noted that for thicker plate bands and a softer foundation (for e.g., *d*/*L* = 0.01, κ = 10^−6^; cf. Figure 10a), this order is disturbed, because for the ratio *E*′′/*E*′ < 0.4, values of these critical forces ratios for plate bands described by function γ(*x*) by (28)_3_ are bigger than those for function γ(*x*) by (28)_2_. 

## 5. Conclusions and Remarks

Summarising considerations and obtained results, the following remarks can be formulated:

- The tolerance modelling approach allows us to replace differential equations of stability with non-continuous, tolerance-periodic, functional coefficients for microstructured functionally graded plates by differential equations with slowly-varying, smooth functional coefficients, which describe the effect of microstructure size on the overall behaviour of these plates.
- The governing equations of the tolerance model for the buckling of microstructured functionally graded plates leads to two formulas of critical forces—the lower fundamental critical force and the additional critical force, so-called the higher critical force.
- Values of the lower fundamental critical forces calculated by the tolerance model are almost the same as those obtained according to the asymptotic model (without the effect of microstructure), but slightly smaller.
- For very thin plate bands on a foundation, differences between the fundamental critical forces obtained in the framework of both the models—tolerance and asymptotic, grow strongly. However, it seems that one problem of this effect is still open.

The proposed tolerance model stands out among the models known from the literature in that it takes into account the effect of microstructure, and moreover, it describes the stability of thin plates with the functional gradation of properties (material and geometric) in the middle plane of the plate, resting on an elastic foundation. It seems appropriate to use this model further to solve specific problems. This study appears to be one of the first concerning the stability of the plates under consideration. In addition, it shows a certain versatility of the tolerance modelling method, which can be used not only in dynamic problems.

The above considerations do not close the subject. It appears that the considered problems will require further analysis, including when it comes to comparing the results obtained numerically or from experimental studies. Some validations of the tolerance model were done for dynamic problems using the finite element method, which was shown, among others in Reference [78] for thin functionally graded plates, and in Reference [72] for periodic Timoshenko beams. The analysis of additional (higher) critical force obtained under the tolerance model also remains open. An analysis of inverse problems will also be interesting in the future; for example, to design the distribution of different materials at the micro-level in order to obtain a specific behaviour of the considered plates.

## Nomenclature

asymptotic model
asymptotic modelling
averaged Euler-Lagrange equations
averaged lagrangean of plate, <Λ*_h_*>
averaged part of in-plane forces, *N*_αβ_
averaging operator, <·
basic cell, ∆
bending stiffnesses of plate, *d*_αβ_ _δ_
dimensionless forces ratios, *n*_−_, *n*_0_
dimensionless microstructure parameter, *l*/*L*
distribution function of material properties, γ(*x*)
effect of microstructure
fluctuation amplitude, *Q^A^*, *Q*
fluctuation shape function, $h^{A},h\in FS_{\delta }^{\alpha }(\Pi ,\Delta )$
functionally graded plate
functionally graded structure
higher critical force, *N*_+_
highly oscillating function, $HO_{\delta }^{\alpha }(\Pi ,\Delta )$
in-plane forces, *n*_αβ_
in-plane forces restriction
lagrangean of plate, Λ
lower critical forces, *N*___, *N*_0_
macrodeflection, *W*
micro-macro decomposition
microstructure parameter, *l*
oscillating part of in-plane forces, ${\tilde{n}}_{\alpha \beta }$
periodic approximation (of function *f*), $\tilde{f}$
plate midplane, Π
Poisson’s ratio of plate material, ν
Ritz method
slowly-varying function, $SV_{\delta }^{\alpha }(\Pi ,\Delta )$
stability of thin microstructured functionally graded plates on an elastic foundation
thickness of plate, *d*
thin microstructured functionally graded plate band
thin microstructured functionally graded plates *aka* tolerance-periodic plates
tolerance averaging approximation
tolerance model
tolerance modelling *aka* the tolerance averaging method
tolerance parameter, δ
tolerance-periodic function, $TP_{\delta }^{\alpha }(\Pi ,\Delta )$
tolerance-periodic structure
Winkler’s coefficient, *k*
Young’s modulus of plate material, *E*.
